# Supplementary figures and images for: Limited initial impacts of biomass harvesting on composition of wood-inhabiting fungi within residual stumps
Source: PeerJ. 2019 Dec 13;7:e8027. doi: 10.7717/peerj.8027 (PMC6913257; doi:10.7717/peerj.8027)

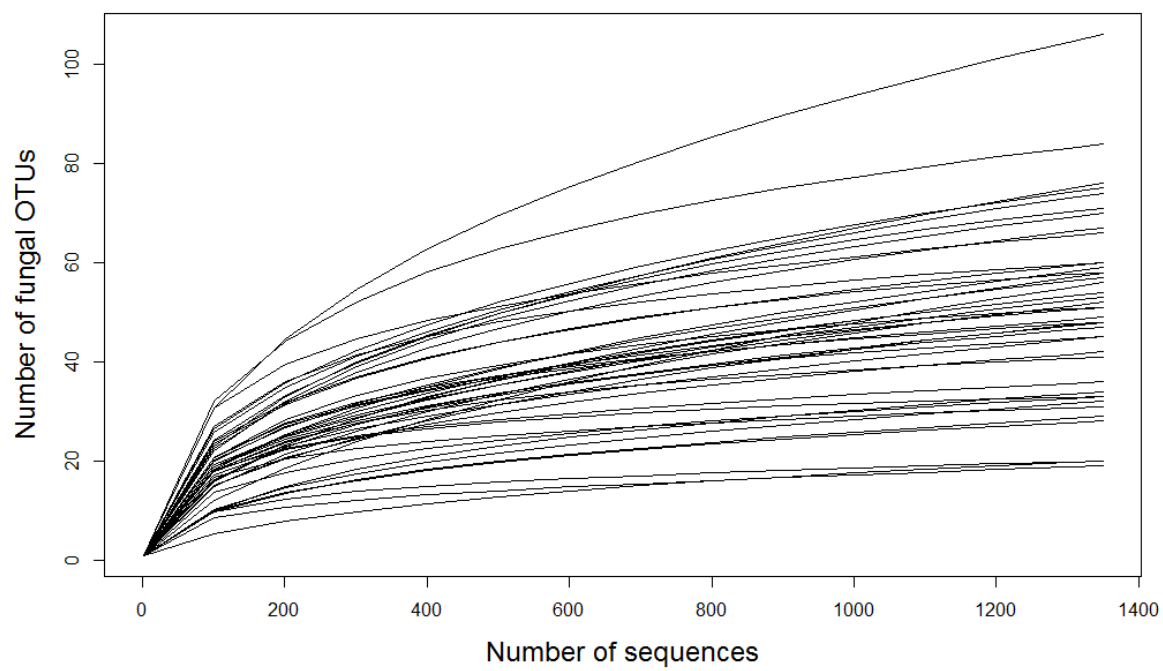

Supplement: Supplemental Information 1 [file peerj-07-8027-s001.pdf]

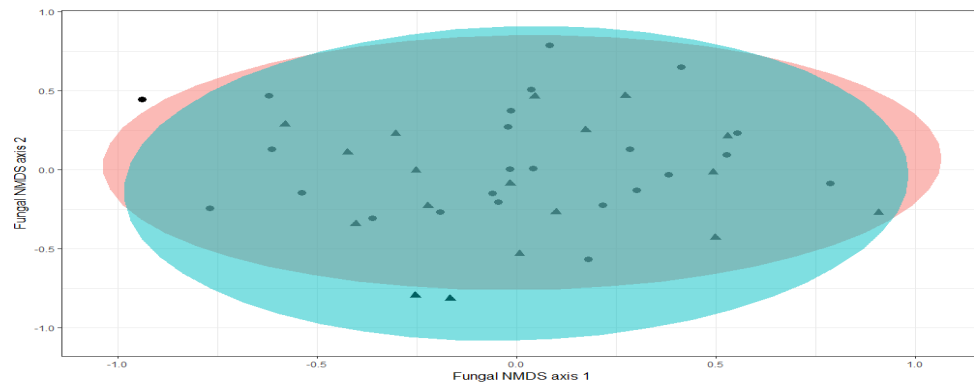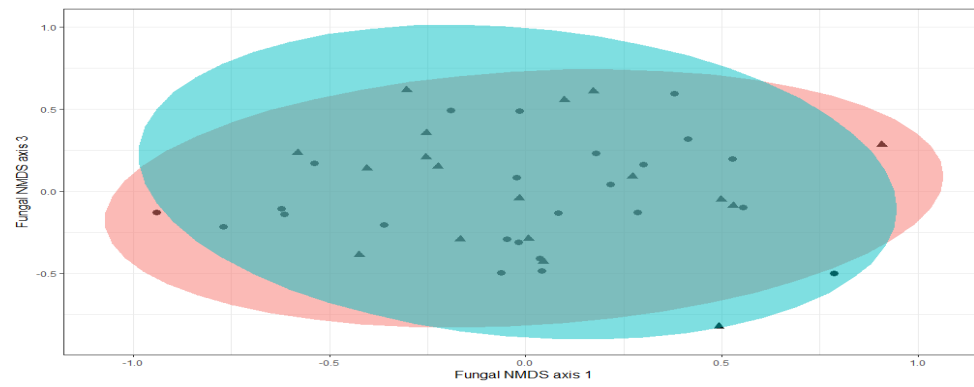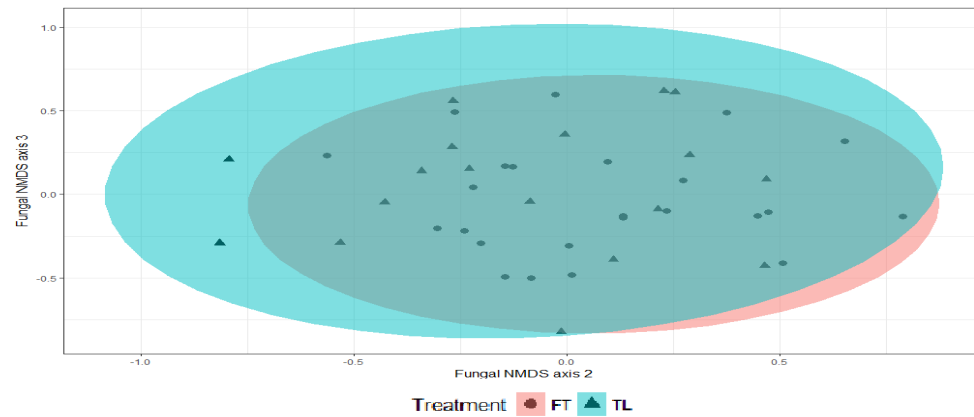

Treatment ● FT ▲ TL

Supplement: Supplemental Information 2 [file peerj-07-8027-s002.pdf]
